# Application of Predictive Modeling and Molecular Simulations to Elucidate the Mechanisms Underlying the Antimicrobial Activity of Sage (*Salvia officinalis* L.) Components in Fresh Cheese Production

**DOI:** 10.3390/foods14132164

**Published:** 2025-06-20

**Authors:** Dajana Vukić, Biljana Lončar, Lato Pezo, Vladimir Vukić

**Affiliations:** 1 Faculty of Technology Novi Sad, University of Novi Sad, Bulevar Cara Lazara 1, 21000 Novi Sad, Serbia; dajanavukic@uns.ac.rs (D.V.); cbiljana@uns.ac.rs (B.L.); 2 Institute of General and Physical Chemistry, Studentski trg 12/V, 11000 Belgrade, Serbia; latopezo@yahoo.co.uk

**Keywords:** antimicrobial potential, sage, extract, inhibitory activity, KdpD histidine kinase

## Abstract

Plant-derived materials from *Salvia officinalis* L. (sage) have demonstrated significant antimicrobial potential when applied during fresh cheese production. In this study, the mechanism of action of sage components against *Listeria monocytogenes, Escherichia coli*, and *Staphylococcus aureus* was investigated through the development of predictive models that describe the influence of key parameters on antimicrobial efficacy. Molecular modeling techniques were employed to identify the major constituents responsible for the observed inhibitory activity. Epirosmanol, carvacrol, limonene, and thymol were identified as the primary compounds contributing to the antimicrobial effects during cheese production. The highest weighted predicted binding energy was observed for thymol against the KdpD histidine kinase from *Staphylococcus aureus*, with a value of −33.93 kcal/mol. To predict the binding affinity per unit mass of these sage-derived compounds against the target pathogens, machine learning models—including Artificial Neural Networks (ANN), Support Vector Machines (SVM), and Boosted Trees Regression (BTR)—were developed and evaluated. Among these, the ANN model demonstrated the highest predictive accuracy and robustness, showing minimal bias and a strong coefficient of determination (R^2^ = 0.934). These findings underscore the value of integrating molecular modeling and machine learning approaches for the identification of bioactive compounds in functional food systems.

## 1. Introduction

One of the main limitations in the production of fresh cheese is its relatively short shelf life, primarily due to its high water content and the absence of a ripening process, which is essential for extending shelf life in ripened cheeses. Antimicrobial properties within the cheese matrix are essential for maintaining food safety and prolonging shelf life by suppressing the growth of harmful bacteria such as *Escherichia coli*, *Staphylococcus aureus*, and *Listeria monocytogenes*. A promising strategy to enhance these antimicrobial features involves the incorporation of food industry by-products. Previous studies explored the use of herbal by-products from filter tea processing, demonstrating their significant antimicrobial potential when applied during fresh cheese production [1]. These plant-derived ingredients exhibited strong inhibitory effects against the aforementioned pathogens, with a notable correlation observed between their total phenolic content and antimicrobial efficacy.

However, the specific phenolic constituents responsible for this activity, as well as their mechanisms of action, remain unclear. Multiple studies have established that essential oils (EO) can exhibit antimicrobial activity through inhibition of ATPase enzymes [2,3]. For instance, limonene has been identified as an ATPase inhibitor in *L. monocytogenes*, with observed damage to the bacterial cell membrane following its application [4]. In addition, compounds such as eugenol, carvacrol, and cinnamaldehyde have been shown to inhibit membrane-bound ATPase activity in *L. monocytogenes* [5]. Further research has demonstrated that exposure to limonene results in reduced Na⁺/K⁺-ATPase and Ca^2^⁺-ATPase activity over a 24 h period [4]. Similar inhibitory effects on ATPase activity have been reported in *S. aureus* treated with EO components such as thymol and carvacrol. These compounds disrupt membrane permeability and interfere with the bacterium’s ATPase function, ultimately impairing its metabolism and growth [6]. Notably, limonene, thymol, and carvacrol were identified in plant extracts used in the production of fresh cheeses [1].

The objective of the present study was to elucidate the mechanism of action of sage-derived antimicrobial compounds against *L. monocytogenes* ATCC 13932, *E. coli* ATCC 8739, and *S. aureus* ATCC 6538 during fresh cheese production by developing predictive models that describe the influence of key parameters on antimicrobial efficacy.

## 2. Materials and Methods

### 2.1. Molecular Modeling

Components of EO and extract obtained by supercritical fluid extraction (SFE) were retrieved from our previous research [1]. In total, 34 compounds were evaluated for their potential antimicrobial activity against *E. coli*, *S. aureus* and *L. monocytogenes* through inhibition of KdpD histidine kinase, a well-known target molecule (Table S1).

Three-dimensional structural models of the KdpD histidine kinase from *E. coli*, *S. aureus* and *L. monocytogenes* were obtained from the AlphaFold Protein Structure Database (AF-P21865, AF-Q2FWH7 and AF-A0A0E1R9J8, respectively) [7]. For subsequent analyses, the catalytic histidine kinase domains were isolated, corresponding to amino acid residues 660–880 in *S. aureus* and 675–892 in *L. monocytogenes*. These domains were structurally and sequentially compared with homologous histidine kinases, including the sensor kinase EnvZ (UniProt: P0AEJ4; PDB ID: 4KP4), in order to identify the binding site. The KdpD histidine kinase from *E. coli* (PDB ID: 6LGQ), although the target enzyme, was not suitable for further analysis due to the absence of the ligand-binding region. EnvZ shares 23.98% sequence identity with *S. aureus* KdpD and 28.93% with *L. monocytogenes* KdpD.

Molecular docking simulations were carried out using Glide 4.0 in extra precision (XP) mode, allowing for ligand flexibility [8]. Docking scores incorporated Epik state penalties to account for protonation state penalties. Binding affinities of the docked ligands were subsequently refined and estimated using the MM-GBSA approach, employing the VSGB 2.0 implicit solvation model [9]. Residues within a 4.0 Å radius of the ligand binding site were treated as flexible to enhance the accuracy of binding mode predictions. The results of the MMGBSA predicted binding energies were weighted (to more realistically reflect the compound’s contribution to antimicrobial activity) by the molecular weight (Da) using the formula: MMGBSA ∗ 100/molecular weight. Molecular dynamics (MD) simulations were performed using the Desmond program incorporated within the Schrodinger suite [10]. The systems were built using the OPLS5 force field and TIP3P solvent model. The system was neutralized by adding Na^+^ ions, and sodium chloride (NaCl) was added to a final concentration of 0.15 M [11]. The simulation was performed using NPT ensemble class and lasted for 200 ns. The final docking poses and protein–ligand interactions were visualized and analyzed using the PyMOL program.

**Figure 1:**
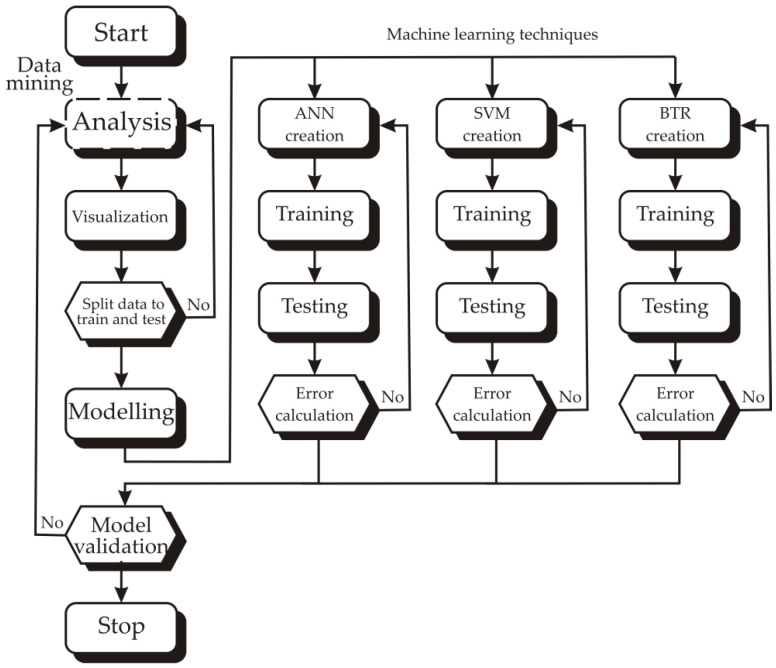
Flowchart of workflow employed in this study, integrating data mining and machine learning techniques for predictive modeling.

The methodology involved a structured workflow encompassing data mining (including analysis and visualization) followed by predictive modeling using three machine learning techniques: Artificial Neural Network (ANN), Support Vector Machines (SVM), and Boosted Trees Regression (BTR) (Figure 1). The dataset was split for training and testing (70% and 30%, respectively). Each model underwent iterative training, testing, and error evaluation. Model performance was then validated to ensure robust and generalizable predictions, culminating in a comparative assessment of the three approaches.

### 2.2. ANN Modeling

For predictive modeling of the antimicrobial mechanism activity of sage components against *L. monocytogenes*, *E. coli* and *S. aureus* during the production of fresh cheese, a three-layer (input, hidden, output) Multi-Layer Perceptron (MLP) ANN was implemented. Given the established suitability of ANNs for addressing non-linear relationships [12,13], both the input variables (bacteria type, compound and binding affinity) and the output variable (binding/weight) were standardized to enhance model accuracy prior to training. During the iterative model building process, input data were consistently fed into the ANN [14,15], and the Broyden–Fletcher–Goldfarb–Shanno (BFGS) algorithm was used for unconstrained non-linear optimization of network parameters.

### 2.3. Support Vector Machine

Support Vector Machine (SVM) models, rooted in averaging principles, are supervised learning algorithms applicable to regression tasks. When employed for regression, SVMs predict outcomes by dividing the dataset into training and testing subsets, making them well-suited for estimating the relationships [16] in nonlinear applications, such as the antimicrobial mechanism activity of sage components *L. monocytogenes*, *E. coli* and *S. aureus* during the production of fresh cheese.

In this study, an SVM model was developed to predict the binding/weight of the input parameters, including bacteria type, compound and binding affinity. This regression type 1 model was configured with a training constant of 10, an epsilon value of 0.1, and a radial basis function with a gamma value of 1.00. The model underwent a total of 20,000 iterations during its training process.

### 2.4. Boosted Trees Regression Model (BTR)

Boosted Regression Tree (BRT) is a sophisticated analytical technique for both prediction and classification tasks, uniquely blending machine learning paradigms with statistical inference. By constructing an ensemble of weak prediction models and iteratively refining them, BRT achieves enhanced predictive power compared to individual models when addressing intricate problems [17,18]. A key strength of BRT lies in its capacity to directly model complex, non-linear patterns within datasets without requiring prior data manipulation, effectively capturing the interplay between predictors and the target variable [19]. This characteristic makes BRT a valuable tool for analyzing natural systems exhibiting complex nonlinearities. The tree-based structure inherent in BRT facilitates visualization and interpretation of the decision-making process. Missing values in predictor variables are handled through surrogate splitting mechanisms within the BRT framework [20]. Moreover, the tree-based nature of BRT contributes to its resilience against the impact of extreme data points. The underlying methodology of BRT involves the application of boosting algorithms, which sequentially build and combine multiple simpler models to achieve superior predictive accuracy, grounded in the principle that combining multiple rough estimates can yield a more accurate final prediction [21,22].

The BTR model was developed and evaluated to predict binding/weight outcomes based on bacteria type, compound, and binding affinity, using the Statistica ver. 10 software, implementation with hyperparameters.

### 2.5. The Models’ Accuracy

To evaluate how well machine learning models could predict output variables based on input data, several statistical tests were calculated. These included the reduced chi-square (χ^2^), root mean square error (RMSE), mean bias error (MBE), mean percentage error (MPE), total squared error (SSE), average absolute relative deviation (AARD), and the coefficient of determination (R^2^). RMSE values help us understand the models’ efficiency by quantifying the agreement between their predictions and the experimental results. Conversely, MBE values indicate the average difference between predicted and observed values [23]. These statistical parameters were calculated based on established equations [24].

(1) $$\chi^{2}=\frac{\sum_{i=1}^{N}{\left(x_{exp,i}-x_{pre,i}\right)}^{2}}{N-n}$$

(2) $$RMSE={\left[\frac{1}{N}\cdot \sum_{i=1}^{N}{\left(x_{exp,i}-x_{pre,i}\right)}^{2}\right]}^{1/2}$$

(3) $$MBE=\frac{1}{N}\cdot \sum_{i=1}^{N}\left(x_{exp,i}-x_{pre,i}\right)$$

(4) $$MPE=\frac{100}{N}\cdot \sum_{i=1}^{N}\left(\frac{\left|x_{exp,i}-x_{pre,i}\right|}{x_{pre,i}}\right)$$

(5) $$SSE=\sum_{i=1}^{N}{\left(x_{exp,i}-x_{pre,i}\right)}^{2}$$

(6) $$AARD=\frac{100}{N}\cdot \sum_{i=1}^{N}\left|\frac{x_{exp,i}-x_{pre,i}}{x_{pre,i}}\right|$$

(7) $$r^{2}=1-\frac{\sum_{i=1}^{N}{\left(x_{exp,i}-x_{pre,i}\right)}^{2}}{\sum_{i=1}^{N}{\left(x_{exp,i}-\bar{x}\right)}^{2}},\bar{x}=\sum_{i=1}^{N}x_{exp,i}$$

where *N* represents the total number of data records, while *x_exp_*_,_*_i_* and *x_pre_*_,_*_i_* are the experimental and model predicted values, respectively.

### 2.6. Statistical Analyses

The normality of the data distribution was evaluated using the Shapiro–Wilk test. Its results showed that most variables did not significantly deviate from normality (*p* > 0.05). Data are expressed as mean values (n = 2). The sample size included 24 samples. Differences between sample means were analyzed using Tukey’s HSD test. The statistical analysis was conducted using the STATISTICA 10.0 software package (StatSoft Inc., Tulsa, OK, USA).

## 3. Results and Discussion

To explore the potential ATPase-inhibitory mechanism underlying the antimicrobial activity of *Salvia officinalis* EO components against *E. coli*, *S. aureus* and *L. monocytogenes*, structural analyses were conducted targeting the KdpD histidine kinase, a recognized antimicrobial drug target. Comparative structural and sequence analyses were conducted between the KdpD histidine kinases from *E. coli*, *S. aureus*, and *L. monocytogenes*, and the histidine kinase EnvZ from *E. coli* (PDB ID: 4KP4), as well as the *E. coli* KdpD crystal structure (PDB ID: 6LGQ), to identify conserved motifs and potential ligand-binding regions. Although KdpD from *E. coli* is directly relevant to the study, its crystal structure lacks the ligand-binding loop, making it unsuitable for molecular docking studies. Consequently, the AlphaFold-predicted structure was employed for subsequent analysis.

### 3.1. Molecular Modeling

**Figure 2:**
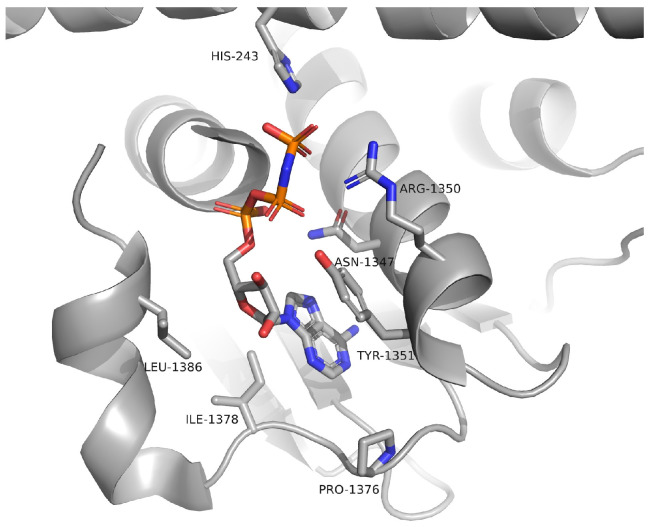
Crystal structure of EnvZ from *E. coli* (PDBID: 4KP4) with ATP bonded in the active site.

**Figure 3:**
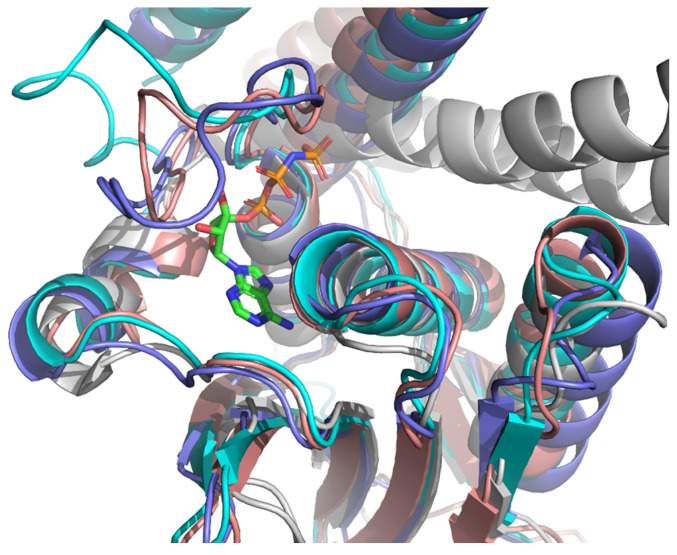
Alignment of crystal structures of EnvZ from *E. coli* (PDBID: 4KP4, gray) with ATP bonded in the active site, KdpD from *E. coli* (AF-P21865, pink), KdpD from *S. aureus* (AF-Q2FWH7, blue) and KdpD from *L. monocytogenes* (AF-A0A0E1R9J8, cyan).

Examination of the EnvZ crystal structure revealed a well-defined ATP-binding and catalytic site (Figure 2), which served as a reference for identifying corresponding regions in the modeled KdpD structures. Comparative analysis confirmed that the catalytic histidine kinase domains of *E. coli*, *S. aureus*, and *L. monocytogenes* KdpD share a high degree of structural similarity with the EnvZ homolog. Furthermore, the AlphaFold models displayed high confidence scores (pLDDT > 70) within the regions corresponding to ATP-binding, supporting their suitability for molecular docking simulations (Figure 3).

To evaluate the potential for inhibiting the targeted KdpD histidine kinases, molecular modeling techniques were applied. The identified bioactive compounds were docked into the active sites of target enzymes, and MMGBSA calculations were performed to calculate binding energies. The results identified several compounds able to inhibit the examined KdpD histidine kinases. The concentrations of the selected compounds in the EO and SFE used for the production of fresh cheese are presented in Table 1, while the results of the molecular modeling simulations are presented in Table 2.

According to the obtained results, thymol exhibited the highest weighted binding energy across all examined *KdpD* histidine kinases. Carvacrol also demonstrated strong binding affinity, particularly toward the *KdpD* histidine kinase from *S. aureus*. These findings are consistent with previous studies that identified thymol and carvacrol as ATPase inhibitors [6]. Limonene exhibited a notably high weighted binding energy against *KdpD* from *L. monocytogenes*, in agreement with earlier reports [5].

**Figure 4:**
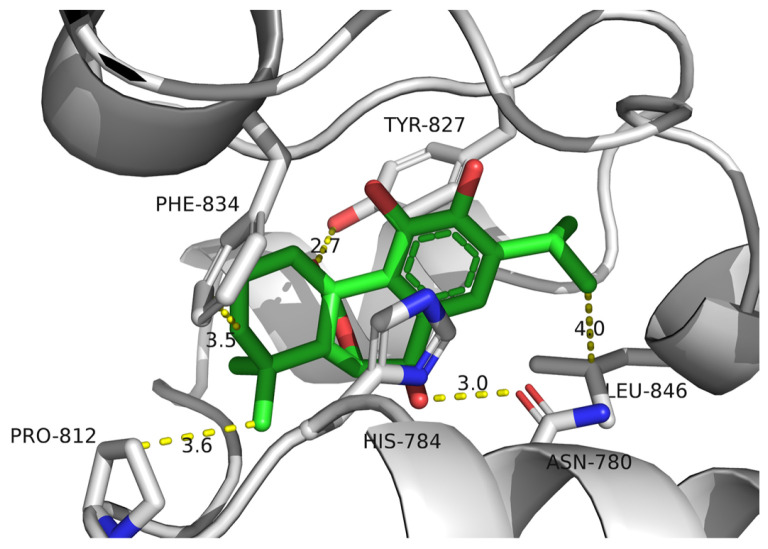
Molecular docking simulation of epirosmanol against KdpD histidine kinase from *S. aureus*: gray—KdpD histidine kinase from *S. aureus*; green—epirosmanol.

**Figure 5:**
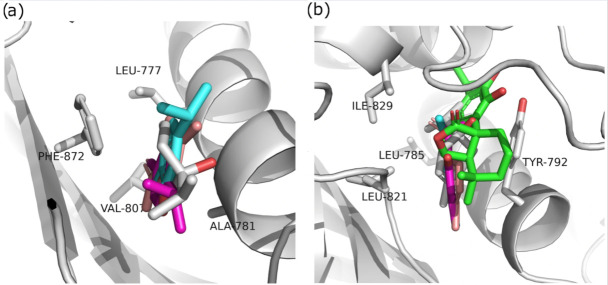
Structural alignment of molecular docking simulations of the selected compounds against (**a**) *KdpD* histidine kinase from *S. aureus*; (**b**) *KdpD* histidine kinase from *E. coli*; carvacrol—cyan, thymol—pink, limonene—red, 4-terpinol—gray, and epirosmanol—green.

**Figure 6:**
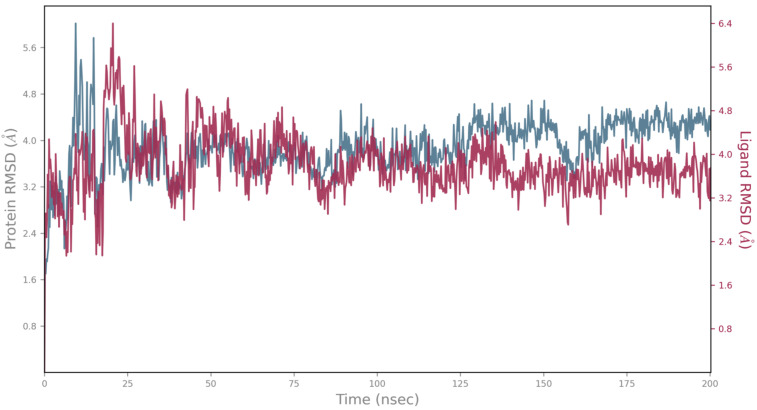
MD simulation of KdpD histidine kinase (*S. aureus*)–epirosmanol complex during 200 ns; blue—protein RMSD; red—ligand aligned on the protein RMSD.

**Figure 7:**
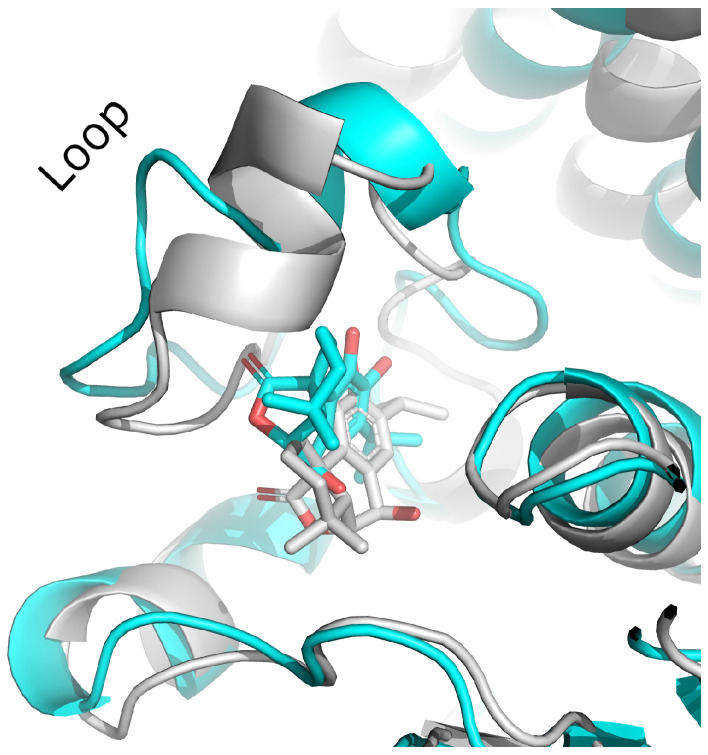
The first and the last frame of the MD simulation of KdpD histidine kinase (*S. aureus*)–epirosmanol complex; gray—first frame; cyan—last frame.

Although these compounds exhibited strong predicted binding affinities toward *KdpD*, their concentrations in EO and SFE were relatively low, suggesting that their contributions may not fully account for the experimentally observed antimicrobial activity (Table 3). Among the compounds present in higher concentrations in EO and SFE, epirosmanol also displayed significant binding affinity, particularly toward the *KdpD* histidine kinase from *S. aureus*. This interaction may play a complementary role in achieving the overall antimicrobial effect observed experimentally. Epirosmanol was found to be stabilized within the *KdpD* binding site through hydrophobic interactions with Pro812 (3.6 Å), Phe834 (3.5 Å), and Leu846 (4.0 Å), which likely contribute to the binding affinity. Stacking interactions with His784, along with polar interactions involving Tyr827 (2.7 Å) and Asn780 (3.0 Å), further enhance the binding specificity (Figure 4). Analysis of other compounds with the potential to bind *KdpD* histidine kinase from *S. aureus* (carvacrol, thymol, limonene, and 4-terpinol) highlighted the crucial role of stacking interactions with His784 and/or Phe872 in combination with deep hydrophobic interactions within the binding pocket (Figure 4 and Figure 5a). Comparative analysis of the *KdpD* histidine kinase from *E. coli* revealed the substitution of His784 with a Tyr residue, which also appeared to play a key role in ligand binding (Figure 5b). In contrast to the *KdpD* histidine kinase from *S. aureus*, the hydrophobic pocket of the *E. coli KdpD* is shallower and located closer to the entrance of the binding cavity. As no previous experimental data are available regarding the ATPase inhibitory activity of epirosmanol, MD simulations were conducted to evaluate the stability of the *KdpD*–epirosmanol complex in *S. aureus*. The simulation results indicate that the complex remains stable over a 200 ns trajectory (Figure 6). Importantly, the protein maintained structural stability during the simulation, particularly within the loop region, validating the suitability of the AlphaFold-predicted structure used in the analysis. The root mean square deviation (RMSD) of the protein–ligand complex closely follows that of the protein itself. While the RMSD initially peaks at 6.4 Å, it is stabilized around 3.5 Å in the latter simulation. This fluctuation was mainly attributed to movement of a loop region involved in ligand binding, as illustrated by the structural alignment of the initial and final MD frames (Figure 7). Although loop dynamics caused minor repositioning of the ligand, the binding interaction remained stable throughout the simulation.

It is important to emphasize that molecular modeling methods, including molecular docking and MD simulations, offer valuable insights into biomolecular interactions, but they come with certain limitations. Although MD simulations are computationally intensive and simulate real conditions, they are limited by the timescales they can realistically cover, potentially missing slow conformational changes. Additionally, the accuracy of these approaches depends on the quality of the input structures and force field parameters. Therefore, future research such as in vitro experiments of inhibitory activity and x-ray crystallography should experimentally confirm these models.

**Figure 8:**
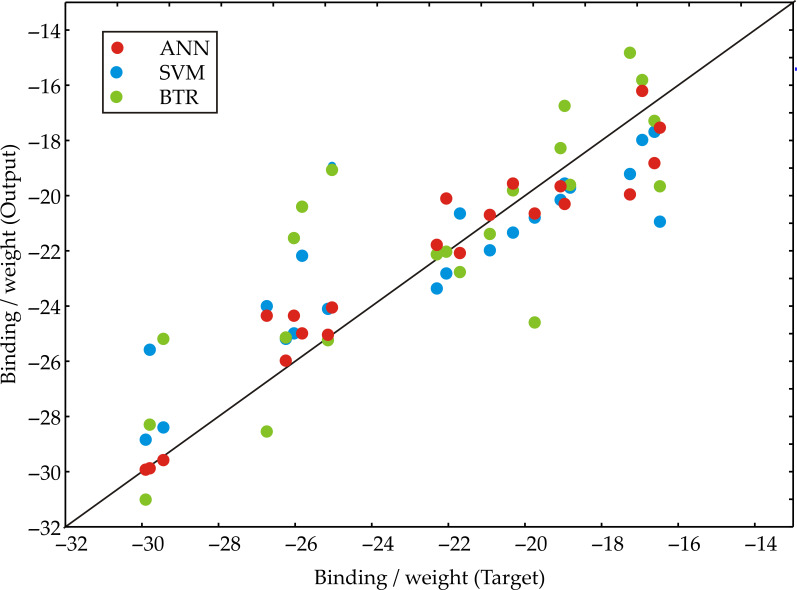
Target vs. prediction for machine learning models.

To identify the components responsible for the obtained experimental results, we developed ANN, Support Vector Machine (SVM), and Boosted Trees Regression (BTR) predictive models. Figure 8 compares the predicted binding affinity per unit weight (Output) against the experimentally determined values (Target) for the three distinct machine learning models. Each data point represents an individual sample, with its color indicating the model used for prediction [25]. The diagonal black line represents perfect agreement between prediction and target, allowing for a visual assessment of each model’s accuracy and any systematic over- or underestimation across the range of binding affinities [17,26]. A tighter clustering of data points around this diagonal line indicates higher predictive accuracy and better model performance.

### 3.2. Artificial Neural Network (ANN) Model

**Figure 9:**
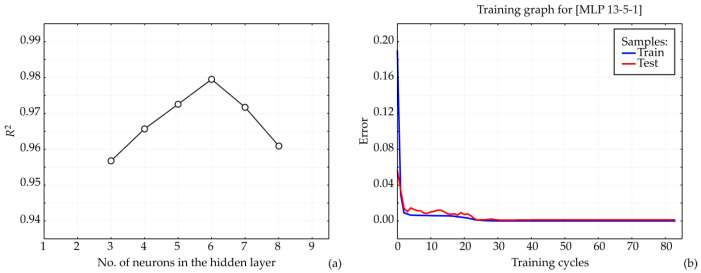
ANN calculations. (**a**) The dependence of the R^2^ value on the number of neurons in the hidden layer in the ANN model, (**b**) training results per epoch.

An Artificial Neural Network (ANN) model was developed in this study, with its architecture and predictive performance strongly influenced by the initial configuration of matrix parameters, including biases and weight coefficients, which are crucial for achieving an accurate fit to the experimental data [27]. The number of neurons in the hidden layer was also found to influence model performance [28]. To mitigate the impact of arbitrary correlations arising from initial parameter assumptions and random weight initialization, each network topology was subjected to 100,000 training iterations [29]. This systematic optimization process revealed that the ANN model achieved its highest coefficient of determination (R^2^) during training when nine hidden neurons were employed (Figure 9).

The ANN regression model (MLP 13-5-1), designed with 13 input neurons, 5 hidden neurons, and 1 output neuron, was used to predict binding/weights based on bacteria type, compound type, and binding type (Table 4).

Table 4 details Table 5 values within matrix *W*_1_ and vector *B*_1_, which is shown as the bias row. Similarly, Table 6 provides the values for matrix *W_2_* and its corresponding bias vector *B*_2_ for the hidden layer computations in the ANN model.

**Table 5:** The weight coefficients and biases *W*_1_ and *B*_1_ for the ANN model.

		1	2	3	4	5
1	Bacteria (*E. coli*)	1.305	0.572	−0.847	0.375	0.519
2	Bacteria (*L. monocytogenes*)	−8.640	−0.765	0.571	−0.577	−6.415
3	Bacteria (*S. aureus*)	6.706	0.950	−0.124	0.575	3.871
4	Compound (4-Terpineol-SFE)	0.498	−0.342	0.276	−0.187	2.083
5	Compound (α-Thujone)	1.760	0.169	−0.013	0.361	0.416
6	Compound (Bornyl acetate)	−7.948	−0.924	1.341	−0.525	−4.416
7	Compound (Carvacrol)	4.683	0.637	−0.737	0.516	3.137
8	Compound (Caryophyllene oxide)	−4.572	−1.169	1.212	−0.797	−4.660
9	Compound (Epirosmanol)	1.558	−0.203	0.394	−0.062	0.459
10	Compound (Limonene)	0.017	1.357	−1.416	0.400	−3.852
11	Compound (Thymol)	3.233	1.226	−1.514	0.620	4.741
12	Binding (No)	0.200	0.539	−0.614	0.256	1.176
13	Binding (Yes)	−0.880	0.261	0.172	0.024	−3.202
	Bias	−0.625	0.815	−0.444	0.311	−2.110

**Table 6:** The weight coefficients and biases *W*_2_ and *B*_2_ for the ANN model.

	1	2	3	4	5	bias
Binding/weight	1.628	−1.995	1.880	−1.237	−7.746	1.230

The model demonstrated strong predictive performance, with a training coefficient of determination (R^2^) of 0.979 and a test R^2^ of 0.991, indicating excellent generalization ability. The training error, calculated using the sum of squares (SOS) error function, was 0.528, while the test error was 2.693. Although validation performance and error metrics were not reported, limiting full assessment of the model’s robustness on truly unseen data, the results suggest minimal overfitting [30]. The model was trained using the Broyden–Fletcher–Goldfarb–Shanno (BFGS) 20 algorithm, which is known for efficient convergence in relatively small networks [31]. A logistic activation function was employed in both the hidden and output layers, indicating that the output values are bounded—appropriate only if the target variable (binding/weight) is normalized or inherently constrained within a 0.000–1.000 range [32]. If the predicted values are continuous and unbounded, the logistic output function may restrict the model’s ability to capture the full output range [33].

The predictions generated by the ANN regression model (MLP 13-5-1) for the variable binding/weight across 24 samples show generally good agreement with the target values, indicating satisfactory model performance. Several cases exhibit minimal deviation, such as case #4 (target: −25.136 vs. predicted: −25.033), case #8 (−29.922 vs. −29.914), case #17 (target: −29.432 vs. 29.567), and case #20 (target: −29.802 vs. −29.884), with absolute differences below 0.1, suggesting high local accuracy. However, some cases show larger discrepancies, including case #2 (target: −22.071 vs. predicted: −20.100), case #3 (−18.967 vs. −20.289), case #11 (−12.823 vs. −15.438), and case #24 (−33.936 vs. −29.911), with differences ranging from approximately 1.3 to 4.0, indicating localized underperformance or deviations potentially linked to underrepresented patterns in the training data. Despite these differences, the model preserves the general trend and magnitude of values, and the prediction errors remain within the acceptable range when considered alongside the overall model performance metrics (training R^2^ = 0.979, test R^2^ = 0.991, training error = 0.528, test error = 2.693).

The residual distribution exhibited a slight right skew, with potential deviation from normality, suggesting the normality assumption may not be fully satisfied [34]. The independence of residuals is presumed valid due to the absence of temporal or spatial ordering in the data. However, the variability of residuals appears to increase for some predicted values, suggesting mild heteroscedasticity, as seen in larger residuals clustered at certain value ranges (e.g., mid-to-low predicted values) [35].

Overall, although the residuals mostly support the reliability of the model, statistical validation indicated that the assumptions of normality and homoscedasticity may be partially violated and warrant formal testing using tools such as the Shapiro–Wilk and Breusch–Pagan tests for comprehensive confirmation [36]. According to the statistical validation of the ANN regression model residuals, the Shapiro–Wilk test for normality gave W = 0.971 and *p* = 0.682, which confirmed that the residuals are normally distributed, as the p-value is well above 0.05. The Breusch–Pagan test for homoscedasticity returned LM = 0.532, *p* = 0.466, suggesting constant variance in the residuals (homoscedasticity), as the *p*-value is also above 0.05.

These results confirm that the residuals satisfy the key assumptions of normality and homoscedasticity, supporting the statistical robustness of the model.

#### Analysis of Influence

**Figure 10:**
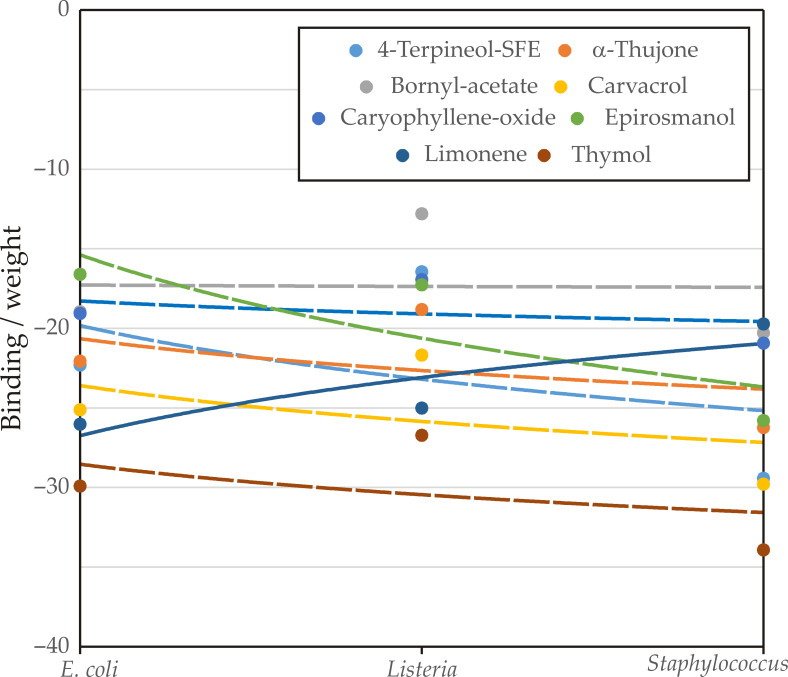
Analysis of influence.

Figure 10 illustrates the binding affinities (expressed as “binding/weight”) of several compounds against *E. coli*, *L. monocytogenes*, and *S. aureus*. For *E. coli*, all compounds except thymol exhibited binding interactions, with limonene showing the strongest affinity (−26.04), followed by carvacrol (−25.14) and thymol (−29.92). Against *L. monocytogenes*, six compounds demonstrated binding, with thymol (−26.73) and limonene (−25.03) again ranking among the most active. For *S. aureus*, seven compounds were able to bind, with thymol showing the highest affinity (−33.94), followed closely by carvacrol (−29.80) and 4-terpineol-SFE (−29.43). These results confirm that thymol, carvacrol, and limonene emerged as the most promising compounds, consistently showing strong binding across multiple bacterial targets.

### 3.3. Support Vector Machine (SVM) Model

The Support Vector Machine (SVM) regression model for predicting binding/weight was constructed using three independent variables, employing a type 1 regression (commonly ε-SVR, which allows for a margin of tolerance around the true values). It utilizes a Radial Basis Function (RBF) kernel, which is effective for capturing nonlinear relationships between predictors and the response variable [37]. The model relies on 15 support vectors, none of which are bounded, meaning that all support vectors contribute meaningfully to defining the regression function without being at the upper/lower margin constraints [38]. This characteristic indicates a well-generalized model with moderate complexity, and suggests that the predictions are not overly influenced by outliers or marginal data points.

SVM model is a type 1 regression model (ε-SVR) with parameters C = 2.0 and ε = 0.1, indicating a moderate tolerance for error (ε) and a regularization strength (C) that balances model complexity with training error. It uses an RBF kernel with γ = 0.333, which enables the model to capture nonlinear relationships between predictors and the target.

The model is defined by 15 support vectors, each associated with a corresponding weight (coefficient). These weights determine the influence of each support vector on the regression function: positive weights (e.g., vector 7: +1.017, vector 14: +1.352) push predictions upward, while negative weights (e.g., vector 15: −1.446, vector 5: −1.166) pull predictions downward.

None of the support vectors are bounded (as noted earlier), indicating that the solution lies well within the feasible region and is not constrained by the penalty term (C), further supporting model stability and generalization.

The decision function of the Support Vector Machine (SVM) regression model includes a constant term (bias) of −0.085, which serves as the intercept in the regression function. This constant is added to the weighted sum of kernel evaluations between the input data and the support vectors.

Given the model specifications, this small bias value indicates that the fitted regression surface is only slightly shifted vertically. The model relies primarily on the support vectors and their associated weights to shape the regression curve, while the decision constant provides a minor adjustment to align predictions with the target values [39]. The small magnitude of this constant suggests that the support vectors collectively provide a strong fit, minimizing the need for a large offset.

Comparing the observed and predicted values across 24 cases revealed that the model captures the general trend and range of the target variable, but exhibits varying levels of prediction error. The predicted values closely match the observed values in some instances—for example, case #11 (observed: −12.82, predicted: −13.88) and case #18 (−26.25 vs. −25.19)—indicating good local approximation. However, larger discrepancies are seen in some cases, such as case #15 (−25.03 vs. −18.93) and case #9 (−16.47 vs. −20.96), suggesting under- or overestimation in certain regions of the feature space.

Overall, the model performs reasonably well but may benefit from further optimization or refinement (e.g., tuning γ or C) to reduce prediction error, particularly in edge cases. The results also underscore the importance of residual analysis and additional validation to ensure robustness across the entire input domain.

Residual analysis of the SVM regression model (C = 2.0, ε = 0.1, RBF kernel with γ = 0.333) shows a mean residual of –0.29 and a standard deviation of 2.20, indicating a slight underestimation tendency. The Shapiro–Wilk test yielded W = 0.896 with *p* = 0.018, suggesting a significant deviation from normality of the residuals. However, the Breusch–Pagan test showed no evidence of heteroscedasticity (LM = 0.276, *p* = 0.599), supporting constant variance across predictions. These results suggest that while the variance assumptions are met, the mild non-normality in the residuals should be addressed for full model robustness.

### 3.4. Boosted Trees Regression Model (BTR)

**Figure 11:**
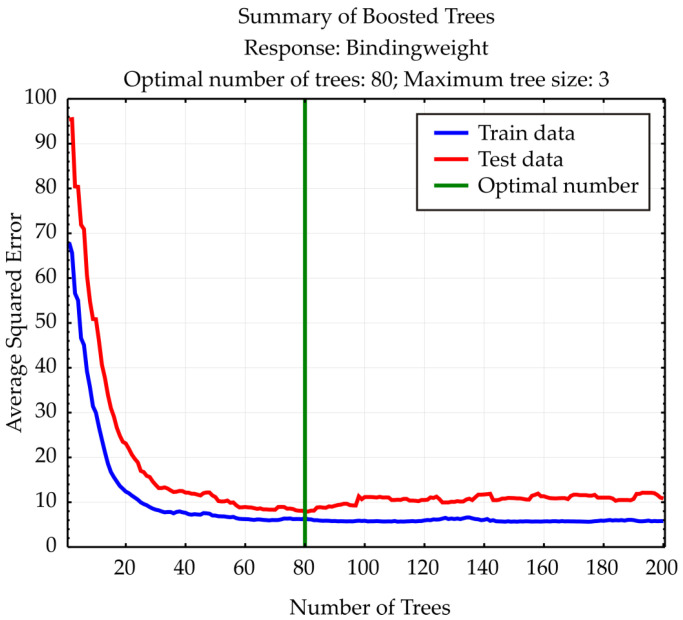
Boosted Trees summary.

The Boosted Trees Regression model, developed using 80 decision trees to predict binding/weight, demonstrated generally strong predictive alignment with the observed values across all samples (Figure 11). Most predicted values are close to their observed counterparts, such as case #1 (observed: –22.32, predicted: –22.12), case #2 (–22.07 vs. –22.05), and case #4 (–25.14 vs. –25.21). However, a few samples show more notable deviations, including case #7 (–26.04 vs. –21.53), case #15 (–25.03 vs. –19.07), and case #23 (–19.74 vs. –24.62), indicating areas where the model under- or over-predicts. The model captures the general trend of the data well but exhibits some sensitivity in outlier regions, suggesting potential room for refinement in tree depth, learning rate, or feature interaction handling.

The residual analysis for the Boosted Trees Regression model indicates satisfactory performance and robustness [40]. The residual mean is approximately −0.60, suggesting a minimal overall bias in predictions. The standard deviation of residuals is 2.53, reflecting the spread of errors around the mean. The Shapiro–Wilk test for normality yielded a *p*-value of 0.448, indicating that the residuals follow a normal distribution (*p* > 0.05).

The Breusch–Pagan test results (LM stat = 0.202, *p* = 0.653) confirmed that the variance of residuals is homoscedastic (constant), as the p-value is well above 0.05.

These outcomes collectively validate the model’s assumptions and suggest that the Boosted Trees Regression model provides consistent and unbiased predictions across the input domain.

### 3.5. Validation of Machine Learning Models

Table 7 displays the verification results of the machine learning models for the observed data.

The analysis clearly indicates that the ANN model yields the most accurate and consistent predictions across the tested sample set. Its low χ^2^ value (2.419) implies a small discrepancy between the observed and predicted values, reinforcing its suitability for modeling binding weights. The RMSE of 1.523 confirms a minimal average prediction error, while the near-zero MBE (−0.072) suggests very little systematic bias. Additionally, the ANN’s negative skewness (−0.371) and moderate kurtosis (0.817) imply a relatively symmetrical error distribution without heavy tails. Its variance (2.414) and standard deviation (1.554) remain within acceptable limits, supporting its predictive stability. In contrast, the SVM model displayed higher residual errors, with RMSE = 2.216 and AARD = 139.379, indicating less precision and greater dispersion. Although SVM’s R^2^ = 0.835 suggests a moderate fit, its larger negative skewness (−0.702) and increased kurtosis (1.266) point to a more skewed and peaked error distribution. The BTR model, with the lowest R^2^ (0.765), showed the greatest deviation from observed values, indicating poorer generalization. Its MPE of −8.646 reflects the largest percentage error among the three models.

Collectively, these metrics confirm that while all three models can predict binding/weights, ANN provides the best overall performance in terms of accuracy, consistency, and residual distribution.

## 4. Conclusions

This study offers a comprehensive mechanistic insight into the antimicrobial potential of *Salvia officinalis* essential oil (EO) and supercritical fluid extract (SFE) components by targeting the KdpD histidine kinase, a key ATPase involved in bacterial signal transduction. Structural modeling, informed by high-confidence AlphaFold predictions and validated through molecular dynamics simulations, confirmed the presence of conserved ATP-binding motifs across KdpD homologs in *E. coli*, *S. aureus*, and *L. monocytogenes*. Molecular docking, MMGBSA binding energy calculations and visual inspection revealed that thymol, carvacrol, and limonene exhibit the strongest binding affinities across multiple bacterial KdpD targets, in alignment with prior reports of their ATPase inhibitory activity. Notably, epirosmanol, despite being present at higher concentrations in the EO and SFE, also demonstrated significant binding affinity and was further validated through a MD simulation, highlighting its stable interaction within the KdpD binding site of *S. aureus.*

To predict antimicrobial efficacy in a practical setting, three machine learning models—ANN, SVM, and BTR—were trained to estimate binding affinity per unit weight of sage constituents. Among them, the ANN model demonstrated the highest predictive performance (R^2^ = 0.934, RMSE = 1.52, minimal bias), outperforming SVM (R^2^ = 0.835) and BTR (R^2^ = 0.765). These findings highlight the effectiveness of machine learning models in capturing the complex relationships between bacteria type, compound, binding affinity, and their resulting binding/weight, suggesting its potential as a valuable tool for predicting antimicrobial interactions in food science, while also underscoring the importance of model selection thorough statistical evaluation.

## Figures and Tables

**Table 1:** Compounds identified in the EO and SFE used for production of kombucha fresh cheese.

Compound	Concentration in EO (%) ^a^	Concentration in SFE (%) ^a^	Molecular Weight (Da)
Epirosmanol	26.25	20.47	346.43
4-Terpineol	-	0.18	154.25
Caryophyllene oxide	0.42	0.63	220.36
Carvacrol	0.95	0.14	150.22
Bornyl acetate	1.26	1.40	196.29
Limonene	0.36	0.51	136.24
α-Thujone	5.26	9.23	152.24
Thymol	1.61	0.40	150.22

^a^ Ref. [1].

**Table 2:** MMGBSA and weighted binding results of the selected, most potent compounds from EO and SFE.

Compound	*E. coli*		*L. monocytogenes*		*S. aureus*	
	MMGBSA dG Bind (kcal/mol)	Binding/ Weight	MMGBSA dG Bind (kcal/mol)	Binding/ Weight	MMGBSA dG Bind (kcal/mol)	Binding/ Weight
Epirosmanol	−57.59	−16.62	−59.84	−17.27	−89.40	−25.80
4-Terpineol	−34.43	−22.32	−25.40	−16.46	−45.40	−29.43
Caryophyllene oxide	−42.04	−19.07	−37.36	−16.95	−46.13	−20.93
Carvacrol	−37.76	−25.13	−32.26	−21.70	−44.77	−29.80
Bornyl acetate	−37.23	−18.96	−25.17	−12.82	−39.85	−20.30
Limonene	−35.48	−26.04	−34.10	−25.03	−26.9	−19.74
α-Thujone	−33.60	−22.07	−28.66	−18.82	−39.96	−26.24
Thymol	−44.95	−29.92	−40.16	−26.73	−50.98	−33.93

**Table 3:** Microbiological profile of the produced kombucha fresh cheese samples (log CFU/g) [1].

Day of Storage	Kombucha C **			Kombucha EO			Kombucha SFE		
	*E. coli*	*L. mono*	*S. aureus*	*E. coli*	*L. mono*	*S. aureus*	*E. coli*	*L. mono*	*S. aureus*
0	3.3 ^a,^*	4.5 ^a^	4.8 ^a^	3.9 ^a^	4.5 ^a^	4.5 ^a^	3.7 ^a^	4.3 ^a^	4.6 ^a^
30	1.5 ^b^	2.6 ^c^	2.9 ^c^	1.0 ^d^	2.5 ^d^	1.9 ^c^	1.9 ^d^	2.5 ^c^	2.4 ^d^

* Values with different letters ^(a–d)^ within each parameter in the same column differ significantly (*p* < 0.05) according to Tukey’s HSD test; ** Kombucha C—kombucha fresh cheese control; Kombucha EO—kombucha fresh cheese with added sage essential oil; Kombucha SFE—kombucha fresh cheese with added sage supercritical extract.

**Table 4:** Summary of ANN model performance.

Net. Name	Training Perf.	Test Perf.	Training Error	Test Error	Training Algorithm	Error Function	Hidden Activation	Output Activation
MLP 13-5-1	0.979	0.991	0.528	2.693	BFGS 20	SOS	Logistic	Logistic

**Table 7:** Verification of models.

	*χ* ^2^	*RMSE*	*MBE*	*MPE*	*SSE*	*AARD*	*R* ^2^	*Skew*	*Kurr*	*Mean*	*StDev*	*Var*
ANN	2.419	1.523	−0.072	−5.559	55.520	33.910	0.934	−0.371	0.817	−0.072	1.554	2.414
SVM	5.124	2.216	−0.289	−7.771	115.836	139.379	0.835	−0.702	1.266	−0.289	2.244	5.036
BTR	7.072	2.603	−0.601	−8.646	153.968	128.476	0.765	−0.348	0.308	−0.601	2.587	6.694

## Data Availability

The original contributions presented in the study are included in the article; further inquiries can be directed to the corresponding author.

## Supplementary Materials

The following supporting information can be downloaded at: https://www.mdpi.com/article/10.3390/foods14132164/s1, Table S1: Compounds identified in the EO and SFE extract used for molecular modeling.
