# Supplementary material for: Application of Predictive Modeling and Molecular Simulations to Elucidate the Mechanisms Underlying the Antimicrobial Activity of Sage (Salvia officinalis L.) Components in Fresh Cheese Production
Source: Foods. 2025 Jun 20;14(13):2164. doi: 10.3390/foods14132164 (PMC12249175; doi:10.3390/foods14132164)
Supplement: Supplementary file 1 [file foods-14-02164-s001.zip › foods-3688521-supplementary.pdf]

Table S1. Compounds identified in the EO and SFE extract used for molecular modeling

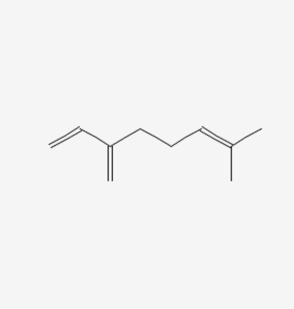<br>**β-Myrcene**	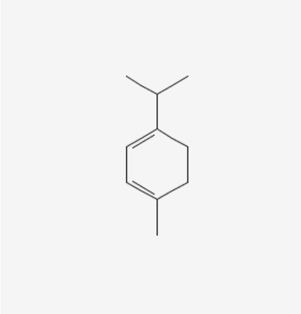<br>**α-Terpinene**	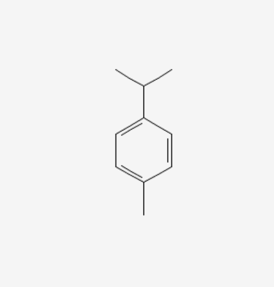<br>**p-Cymene**	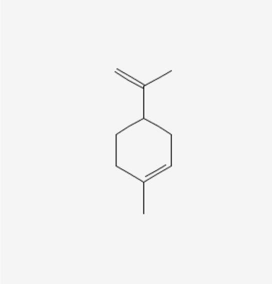<br>**Limonene**
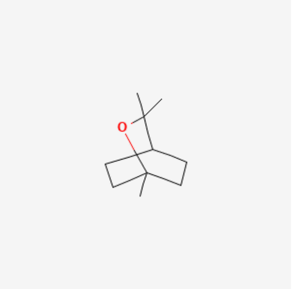<br>**Eucalyptol (1,8-Cineol)**	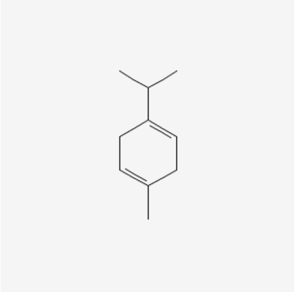<br>**γ-Terpinene**	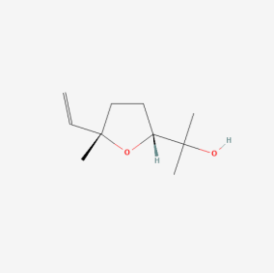<br>**cis-Linalool oxide**	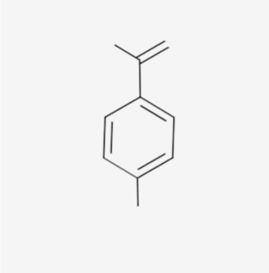<br>**Dehydro-p-cymene**
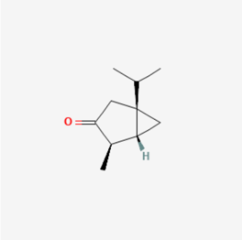<br>**α-Thujone**	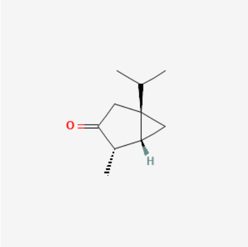<br>**β-Thujone**	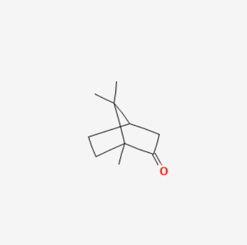<br>**Camphor**	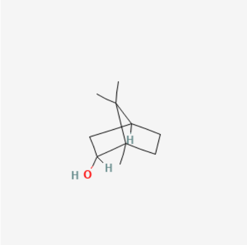<br>**Borneol**
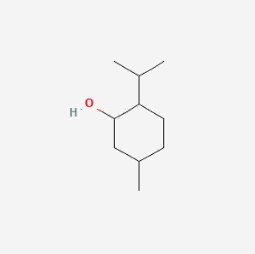<br>**Menthol**	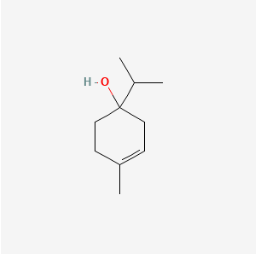<br>**4-Terpineol**	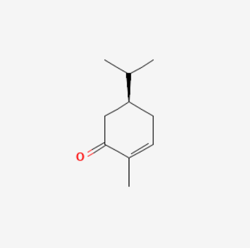<br>**Carvotanacetone**	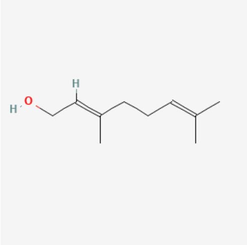<br>**trans-Geraniol**
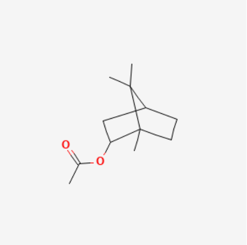<br>**Bornyl acetate**	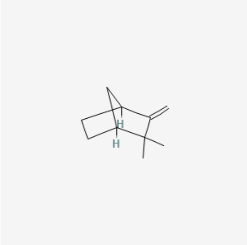<br>**Camphene**	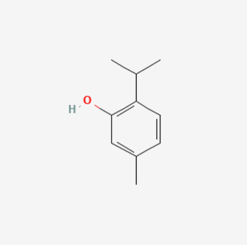<br>**Thymol**	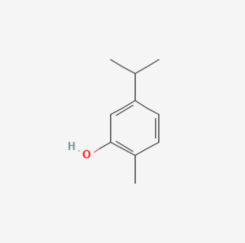<br>**Carvacrol**

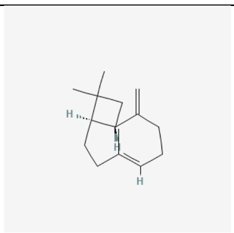<br>**γ-Caryophyllene**	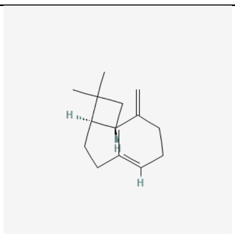<br>**trans-Caryophyllene**	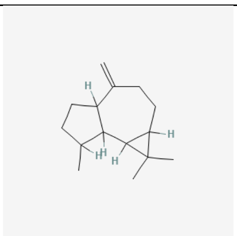<br>**Aromadendren**	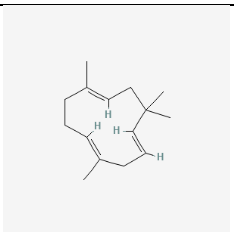<br>**α-Humulene**
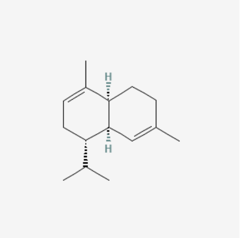<br>**Ledene**	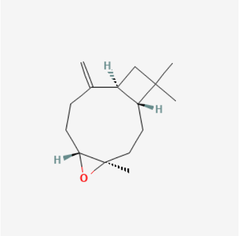<br>**Caryophyllene oxide**	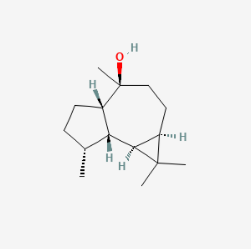<br>**Viridiflorol**	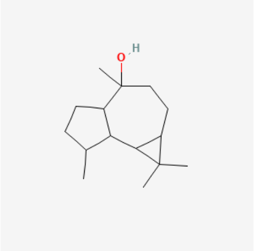<br>**Ledol**
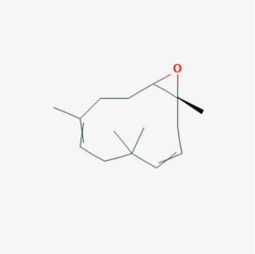<br>**Humulene oxide**	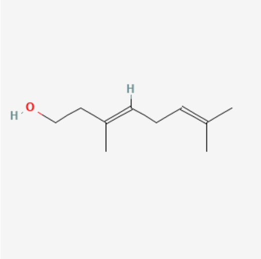<br>**iso-Geraniol**	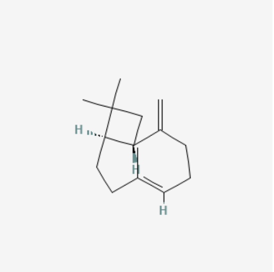<br>**Caryophyllene**	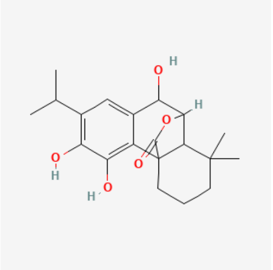<br>**Epirosmanol**
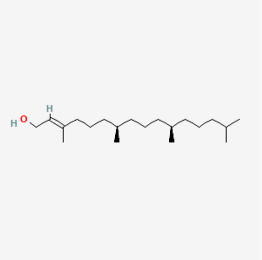<br>**Phytol**	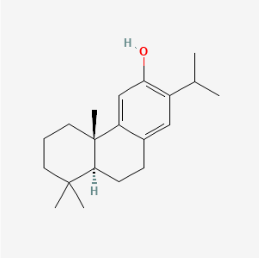<br>**Ferruginol**		
